# Utility of Low-Dose Duvelisib for Advanced Mycosis Fungoides: A Single-Institution Study

**DOI:** 10.1093/oncolo/oyad345

**Published:** 2024-01-18

**Authors:** Christopher Bazewicz, Nicolena Verardi, Oleg Akilov

**Affiliations:** Department of Dermatology, Penn State University, Hershey, PA, USA; Cutaneous Lymphoma Program, University of Pittsburgh, Pittsburgh, PA, USA; Cutaneous Lymphoma Program, University of Pittsburgh, Pittsburgh, PA, USA

**Keywords:** cutaneous T-cell lymphoma, mycosis fungoides, duvelisib, PI3K-δ,γ inhibitor

## Abstract

Duvelisib, a small-molecule phosphatidylinositol 3-kinase-δ,γ inhibitor, has shown efficacy for mycosis fungoides (MF) at dosage ranges of 25-100 mg twice daily (BID), but with significant toxicity. We conducted a retrospective cohort study of patients with advanced MF treated with low-dose duvelisib (15 mg every other day to BID), in an effort to minimize toxicity. A total of 7 patients were included. The overall response rate on duvelisib was 71%, with the remaining patients maintaining stable disease. Mean modified Severity Weighted Assessment Tool score improved by 57.4% and mean percent body surface area involved improved by 52%. Median progression-free survival was 10.3 months. Adverse events occurred in 4 of 7 patients, the most common being fatigue (2/7; grades 1-2), nausea (2/7; grades 1-2), and transaminitis (2/7; grade 3). Overall, low-dose duvelisib showed efficacy for advanced MF with less toxicity, providing a rationale for its use as monotherapy and potentially combinatorial therapy.

## Teaser

Management of advanced mycosis fungoides poses significant challenges, and novel therapeutic approaches are needed. This article reports efforts to minimize toxicity using a lower dosage range of duvelisib.

The management of advanced mycosis fungoides (MF) poses significant challenges, necessitating the exploration of novel therapeutic approaches. Current systemic treatments are limited and include oral retinoids, interferons, brentuximab vedotin, mogamulizumab, extracorporeal photopheresis, pralatrexate, histone-deacetylase inhibitors, and single or multiagent chemotherapy.^1^ None of these modalities are used with an intention to cure, thus, ongoing efforts to discover effective systemic treatments for MF remain. One potential option is duvelisib, a small-molecule phosphatidylinositol 3-kinase (PI3K)-δ,γ inhibitor, which is approved for chronic lymphocytic leukemia/small lymphocytic lymphoma.^2^ Specifically, a recent phase I trial demonstrated the efficacy of duvelisib in peripheral T-cell lymphomas and cutaneous T-cell lymphomas, including MF. However, significant toxicity was observed when dosing ranged from 25 to 100 mg twice daily (BID).^3^ In this report, we present our experience with a lower dosage range of duvelisib, aimed at minimizing toxicity, for advanced MF.

We conducted a single-institution retrospective cohort study analyzing the outcomes of patients with advanced MF treated with duvelisib between March 2022 and June 2023. We included adult patients with MF who had tumor-stage disease at some point in their disease course. Initial duvelisib dosing was 15 mg daily with subsequent dose modification based on patient weight (eg, increase to 15 mg BID was considered for a weight >90 kg) and tolerability. Doses ultimately ranged from 15 mg once every other day to BID. One patient received 25 mg once daily duvelisib (due to the patient’s phenobarbital decreasing duvelisib absorption). Patients were allowed to receive concomitant skin-directed and systemic therapies to mirror real-world MF management. We used the modified Severity Weighted Assessment Tool (mSWAT) to evaluate treatment response.^4^ A partial response (PR) was defined as ≥50% improvement in the mSWAT from baseline, while progressive disease (PD) was ≥25% worsening in the mSWAT.^4^ Stable disease (SD) was an mSWAT that remained between these 2 values, and complete response (CR) was 100% disease clearance.^4^ Relapse was defined as a loss of response during or after treatment with duvelisib. The best response in mSWAT, percent body surface area (%BSA), and each compartment (skin, lymph nodes, viscera, and blood) when applicable, were collected, as well as time to response (TTR), duration of response, and median progression-free survival (PFS). Adverse events (AEs) to duvelisib and reasons for discontinuation were collected. AEs were assessed by laboratory testing and clinical evaluation at each visit. AE severity was graded using the National Cancer Institute Common Terminology Criteria for AEs version 4.03. Causal relationships between AEs and duvelisib were investigator-determined based on the known AE profile of duvelisib.

A total of 7 patients were included, and baseline demographics and disease characteristics are summarized in Table 1. The average age at MF diagnosis was 50 years and the average duration of disease prior to the study was 16 years. The majority of patients were White (71%) and male (57%). All patients had advanced stage MF (stage ≥ IIB) at some point in their disease course. At the study initiation, the majority of patients (71%) had stage IIB disease. The average age at the time of duvelisib initiation was 66 years. The baseline mean mSWAT was 48 and baseline mean %BSA affected was 29%.

**Table 1.** Baseline demographics and disease characteristics.

Baseline demographics	
Age (years) at diagnosis, mean (range)	50 (27-70)
Sex, male, *n* (%)	4 (57%)
Race/ethnicity (*n*)	White (5) Black (2)
Disease duration (years) prior to duvelisib, mean (range)	16 (2-27)
Worst clinical stage prior to duvelisib (*n*)	IIB (6) IVB (1)
Number of prior skin-directed and systemic treatments, median (range)	7 (3-16)
Age (years) at time of duvelisib initiation, mean (range)	66 (44-79)
MF TNMB stage at the beginning of duvelisib (*n*)	T2N0M0B0 (2) T3N0M0B0 (4) T3N1M0B0 (1)
MF clinical stage at the beginning of duvelisib (*n*)	IB (2) IIB (5)
%BSA at the beginning of duvelisib, mean (range)	29% (range 6%-88%)
mSWAT at the beginning of duvelisib, mean (range)	48 (range 9-123)

Abbreviations: MF, mycosis fungoides; TNMB, tumor-node-metastasis-blood; %BSA, percent body surface area; mSWAT, modified Severity Weighted Assessment Tool.

**Figure 1.**
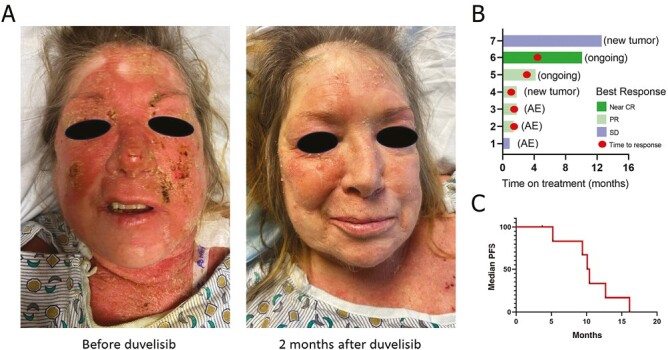
Clinical responses to duvelisib. (**A**) Representative images of clinical improvement on duvelisib 2 months after initiation of therapy. (**B**) Responses to duvelisib and reasons for discontinuation of therapy in our patient cohort. (**C**) Median progression-free survival of our patient cohort. Abbreviations: AE, Adverse event; CR, complete response; PR, partial response; SD, stable disease; PFS, progression-free survival.

Responses to duvelisib and representative images are presented in Fig. 1. Concomitant skin-directed and systemic therapies during duvelisib treatment are summarized in Supplementary Material Table S1. The overall response rate on duvelisib was 71% (1 near CR; 4 PR), while the remaining patients maintained SD. No patient experienced PD during the study (Fig. 1B). On average, patients received duvelisib for 4.7 months (range of 28-377 days). During duvelisib treatment, mean mSWAT improved by 57.4% (range of 18.6%-94.3%) and mean %BSA improved by 52% (range of 20.2%-91.9%). Among partial responders, the average TTR was 2.5 months (range of 42-133 days) and the average duration of response was 4.2 months (range of 22-290 days). Two of 5 patients (40%) maintained a PR at the time of data analysis and continued on duvelisib. The remaining 3 partial responders (60%) experienced relapse after discontinuation of duvelisib. Complete details regarding the duration of response and time to relapse are presented in Supplementary Material Table S2. The median PFS for the patient cohort was 10.3 months (Fig. 1C).

AEs to duvelisib are presented in Supplementary Material Table S3. AEs occurred in 4 of 7 patients (57%). The most common AEs included fatigue (2/7; grade 1-2), nausea (2/7; grade 1-2), and transaminitis (2/7; both grade 3). One patient developed severe neutropenia, skin infection, bacteremia, and sepsis, necessitating therapy discontinuation. The patient died several months later, possibly hastened by the myelosuppressive effects of duvelisib. Transaminitis and nausea, vomiting, and colitis were the reasons for therapy discontinuation in 2 patients. Of note, one patient with grade 3 transaminitis responded to a 1-month drug holiday and prednisone taper and tolerated a lower dose of duvelisib with no further AEs. Five of 7 patients (71%) ultimately discontinued treatment. Reasons for duvelisib discontinuation included the AEs (3/7) noted above, which generally occurred on 15 mg daily to BID dosing, and the development of new tumors (2/7; Fig. 1B).

In this single-institution retrospective study, a lower-dose of duvelisib showed efficacy for advanced MF. In comparison to data published by Horwitz et al,^3^ our study expands on the use of duvelisib for the treatment of advanced MF, using a lower dosage range of duvelisib to minimize toxicity. Horwitz et al treated patients with MF with duvelsib 25-100 mg BID and reported an overall response rate of 31.6% with no CR and a median PFS of 4.5 months. Our patients received 15 mg duvelisib every other day to BID; we observed an overall response rate of 71% with 1 near CR and a median PFS of 10.3 months. For further comparison, in the MAVORIC trial, the median PFS for patients with tumor-stage MF (5 patients in our cohort) receiving mogamulizumab was 4.2 months and 3.9 months for vorinostat.^5^ AEs lead to discontinuation of therapy in 3/7 patients in our study, underscoring the need for appropriate patient selection, patient counseling, and laboratory monitoring.

Limitations of our study include a small sample size, which may restrict the generalizability of our findings. Additionally, the uncontrolled nature and concomitant use of skin-directed and systemic therapies limits the ability to determine the precise impact of duvelisib on disease response. Further, our patient cohort primarily had skin-limited disease, thus research is needed to investigate the efficacy of duvelisib in patients with systemic involvement. Future studies should be designed with these considerations in mind, as well as exploring potential drug combinations. Such investigations would provide valuable insights into the utility of duvelisib for MF. Nevertheless, the results of our study suggest that a lower-dose of duvelisib may be a useful systemic therapy, alone or in combination with skin-directed therapies or other systemic therapies, for the treatment of clinical stage IB to IIB MF.

## Supplementary Material

Supplementary material is available at *The Oncologist* online.

oyad345_suppl_Supplementary_Table_1

oyad345_suppl_Supplementary_Table_2

oyad345_suppl_Supplementary_Table_3

## Data Availability

The data underlying this article will be shared on reasonable request to the corresponding author.
