# Supplementary material for: Utility of Low-Dose Duvelisib for Advanced Mycosis Fungoides: A Single-Institution Study
Source: Oncologist. 2024 Jan 18;29(3):272–4. doi: 10.1093/oncolo/oyad345 (PMC10911911; doi:10.1093/oncolo/oyad345)
Supplement: oyad345_suppl_Supplementary_Table_1 [file oyad345_suppl_supplementary_table_1.docx]

**Supplementary Table 1.** Concomitant skin-directed and systemic therapies during treatment with duvelisib^a^

| Patient (stage at duvelisib initiation) | Concomitant radiation and systemic therapy |
| --- | --- |
| Patient 1 (IIB; T3N1M0B0) | None |
| Patient 2 (IIB; T3N0M0B0) | Nivolumab 480 mg IV (1 dose), romidepsin 14 mg/m2 every other week (1 dose), 20 mg/day prednisone (chronic medication) |
| Patient 3 (IIB; T3N0N0B0) | None |
| Patient 4 (IB; T2N0M0B0) | None |
| Patient 5 (IB; T2N0M0B0) | Targeted radiation |
| Patient 6 (IIB; T3N0M0B0) | Targeted radiation |
| Patient 7 (IIB; T3N0M0B0) | Nivolumab 480 mg IV (1 dose), romidepsin 14 mg/m2 every other week (1 dose), bexarotene 150 mg/day (8 months), targeted radiation |

^a^All patients in our cohort had topical steroids to use as needed for the treatment of their mycosis fungoides.

TNMB, tumor-node-metastasis-blood
