# Supplementary material for: Utility of Low-Dose Duvelisib for Advanced Mycosis Fungoides: A Single-Institution Study
Source: Oncologist. 2024 Jan 18;29(3):272–4. doi: 10.1093/oncolo/oyad345 (PMC10911911; doi:10.1093/oncolo/oyad345)
Supplement: oyad345_suppl_Supplementary_Table_2 [file oyad345_suppl_supplementary_table_2.docx]

**Supplementary Table 2.** Duration of response and time to relapse

| Patient (stage at duvelisib initiation) | Best response | Duration of response and time to relapse (days) |
| --- | --- | --- |
| Patient 1 (IIB; T3N1M0B0) | SD | N/A |
| Patient 2 (IIB; T3N0M0B0) | PR | 22^a^ |
| Patient 3 (IIB; T3N0N0B0) | PR | 78^b^ |
| Patient 4 (IB; T2N0M0B0) | PR | 290^c^ |
| Patient 5 (IB; T2N0M0B0) | PR | 66^d^ |
| Patient 6 (IIB; T3N0M0B0) | Near CR | 171^d^ |
| Patient 7 (IIB; T3N0M0B0) | SD | N/A |

^a^Following duvelisib, the patient remained on 20 mg/day prednisone and topical steroids at the time of relapse.

^b^Following duvelisib, the patient was receiving targeted radiation and topical steroids at the time of relapse.

^c^Following duvelisib, the patient received targeted radiation, 4 doses of brentuximab vedotin (1.8 mg/kg), 2 doses of 200 mg IV pembrolizumab and was on topical steroids prior to relapse.

^d^Both patients maintained their best responses at the time of data analysis.

TNMB, tumor-node-metastasis-blood; SD, stable disease; PR, partial response; CR, complete response; N/A, not applicable
