# Supplementary material for: Utility of Low-Dose Duvelisib for Advanced Mycosis Fungoides: A Single-Institution Study
Source: Oncologist. 2024 Jan 18;29(3):272–4. doi: 10.1093/oncolo/oyad345 (PMC10911911; doi:10.1093/oncolo/oyad345)
Supplement: oyad345_suppl_Supplementary_Table_3 [file oyad345_suppl_supplementary_table_3.docx]

**Supplementary Table 3.** Adverse events on duvelisib

| Adverse event | Frequency, n | Grade |
| --- | --- | --- |
| Fatigue | 2/7 | 1 and 2 |
| Transaminitis^a^ | 2/7 | 3 |
| Nausea | 2/7 | 1 and 2 |
| Vomiting | 1/7 | 1 |
| Belching | 1/7 | 1 |
| Colitis | 1/7 | 2 |
| Fever | 1/7 | 1 |
| Neutropenia^b^ | 1/7 | 4 |
| Bacteremia^b^ | 1/7 | 2 |
| Skin infection^b^ | 1/7 | 3 |
| Myalgia | 1/7 | 2 |

^a^One patient with grade 3 transaminitis responded well to a 1-month drug holiday and prednisone taper, and tolerated lower dose duvelisib with no further adverse events.

^b^One patient developed severe neutropenia, skin infection, bacteremia and sepsis, necessitating therapy discontinuation. The patient ultimately died several months later, possibly hastened by the myelosuppressive effects of duvelisib.
